# SMiRK: an Automated Pipeline for miRNA Analysis

**Published:** 2015-06

**Authors:** Brandon Milholland, Saurabh Gombar, Yousin Suh

**Affiliations:** Albert Einstein College of Medicine, 1300 Morris Park Avenue, Bronx, NY10461, USA

## Abstract

**Background:**

Micro RNAs (miRNAs), important regulators of cell function, can be interrogated by high-throughput sequencing in a rapid and cost-effective manner. However, the tremendous amount of data generated by such methods is not easily analyzed. In order to extract meaningful information and draw biological conclusions from miRNA data, many challenges in quality control, alignment, normalization, and analysis must be overcome. Typically, these would only be possible with the dedicated efforts of a specialized computational biologist for a sustained period of time.

**Results:**

Here, we present SMiRK, an automated pipeline that allows such tasks to be completed with minimal time and without dedicated bioinformatics personnel. SMiRK’s flexibility also allows experienced users to exert more control, if they wish. We describe how SMiRK automatically normalizes the data, removes low-information miRNAs, and produces heatmaps of the processed data. We give details on SMiRK’s implementation and use cases for novice and advanced users. As a demonstration of its capabilities, SMiRK was used to rapidly and automatically analyze a dataset taken from the literature.

**Conclusion:**

SMiRK is a useful and efficient tool that can be used by investigators at multiple skill levels. Those who lack bioinformatics training can use it to easily and automatically analyze their data, while those with experience will find it beneficial to not need to write tools from scratch.

## Introduction

Since their discovery, micro RNAs (miRNAs)—small RNA molecules of 18–25 bp that post-transcriptionally regulate gene expression—have been increasingly recognized as key mediators of a wide range of biological processes in humans and other organisms [1–8]. High throughput analysis of miRNAs, originally accomplished through microarray technology, has given way to sequencing analysis for several reasons. These reasons include: miRNAs are fewer in number and smaller in size than most other RNA species, and they require less sequencing capacity than conventional transcriptome studies. This means that indexed libraries from many samples can be simultaneously sequenced on a single lane on a high-throughput platform like the Illumina HiSeq 2500 or Ion Torrent Proton. As a result, miRNA sequencing is a useful tool for studies in which many samples are collected.

The utility of miRNA sequencing in producing large amounts of data is diminished by the difficulties of data analysis. Necessary steps after sequencing include: alignment of the raw data to known miRNA sequences, mathematical normalization of quantitative read counts, and determination of significant differences between each experimental group. Typically, these tasks require specialized knowledge and computational skills, which necessitate dedicating informatics and statistics personnel to the analysis. Furthermore, the complexity of these tasks can often cause them to take weeks or even months to complete, causing a bottleneck in the scientific process that is inconsistent with the speed with which data can be produced.

In order to solve the problems presented by the analysis of miRNA sequence data, we have developed an automated pipeline called SMiRK. This pipeline takes care of the major tasks of miRNA sequence data analysis; it can be easily run by investigators who do not have access to informatics cores. Furthermore, since it is automatic, running SMiRK requires only a small amount of active time on the part of the user.

It is possible that for some use cases, however, SMiRK’s default workflow is not appropriate; for that reason SMiRK’s individual modules can also act as standalone tools, which can assist users who wish to perform bespoke analyses.

## Implementation

**Figure 1:**
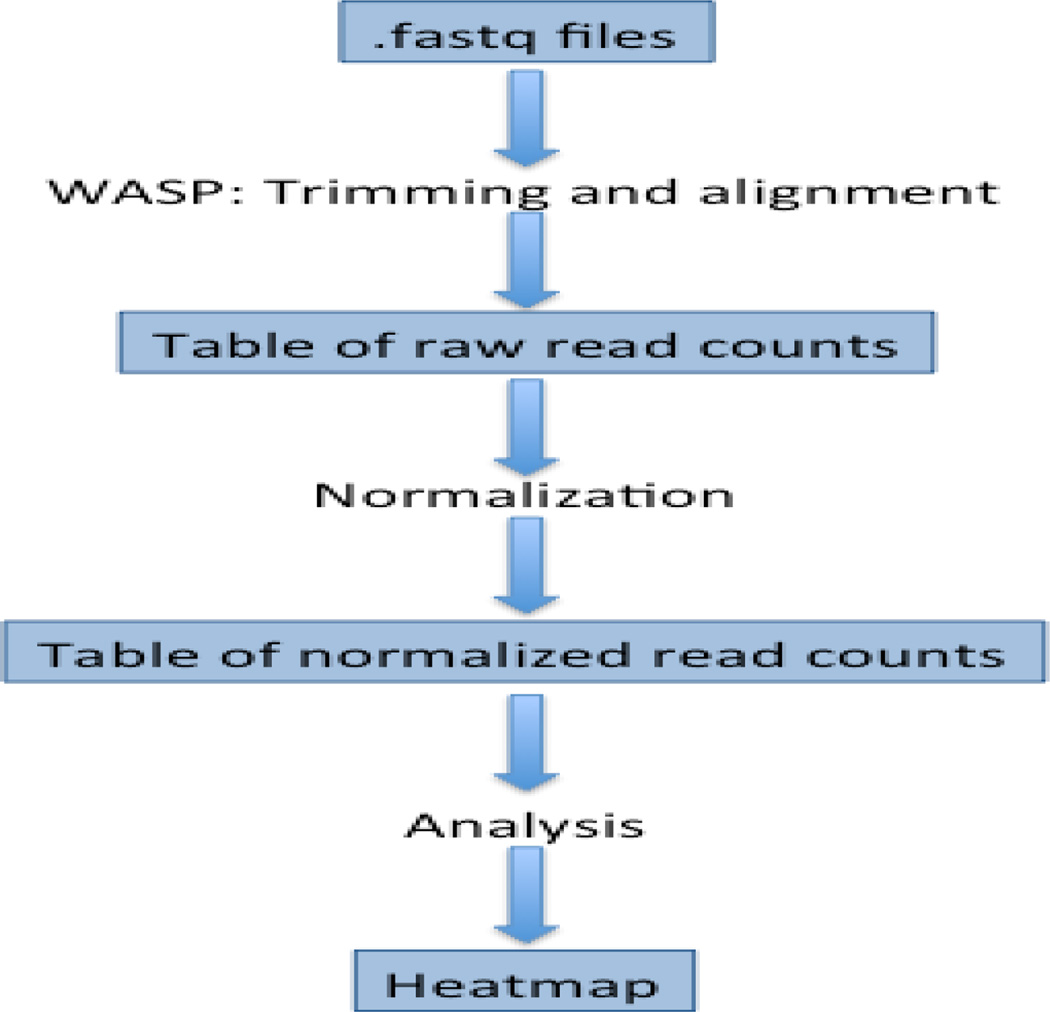
Outline the of SMiRK process.

SMiRK is implemented in the form of several modules, which perform the tasks of: adaptor trimming, alignment, normalization, removal of low-abundance miRNAs, and analysis (Figure 1). sequence data. The WASP system is used to trim the adaptors from the sequences and align them to miRNA sequences. The resulting table of miRNA read counts is normalized by the rpm method, producing a table of normalized read counts. Finally, the expression levels of miRNAs are visualized on a heatmap.

First, raw files, in the FASTQ format, must have their adaptors trimmed. Then, the trimmed reads are aligned with the mature miRNA sequences in version 20 of the relevent mirBase database [9] for the species using Bowtie [10] with the best and tryhard parameters. The result is a table of miRNA read counts for each library. SMiRK was designed to use output from the Wiki-Based Automated Sequence Processor (WASP) [11,12] implementation of these steps. SMiRK, however, is versatile, and can accept as input a comma-separated table of miRNA counts from any source.

Next, read counts must be normalized between libraries. Depending upon sample quality and quantity, library preparation protocol, accuracy of quantification prior to sequencing and quality of the final sequence, the total read counts can vary dramatically between libraries. If this is not accounted for, results can be greatly altered, and both false positives and false negatives can result. For example, if one library has many more reads than another, then miRNAs in that sample may appear to be overexpressed, leading to a false positive. On the other hand, if two libraries from the same group have vastly different numbers of reads, then the expression of miRNAs in that group may appear to be highly variable, which will reduce the power of any subsequent statistical tests and lead to a risk of false negative results. Therefore, normalization of library read counts is essential if any comparisons are to be made of miRNA expression levels between libraries.

Library normalization is implemented in SMiRK by a custom-built Java program. The normalization is based on the reads per million (rpm) method [13]. With this method, the number of reads aligned to each individual miRNA in a library is divided by the total number of miRNA aligned reads in that library, and then multiplied by 1,000,000. The result is that each library is normalized to a size of one million reads; and because each library has effectively the same size, the expression level of any given miRNA can be compared between multiple libraries. After normalization, it is often apparent that many miRNAs are completely unexpressed or expressed at very low abundance in all samples. Since they do not contain any meaningful information about the differences between samples, further analysis is simplified by removing them entirely. This step is implemented by a Python-based program that removes low-abundance miRNAs (defined as miRNAs with a count of fewer than 10 reads per million in more than half of the samples).

Finally, SMiRK performs analysis on the normalized data. After alignment and normalization, the resulting data can be highly informative and useful; however, the information is still relatively inaccessible since a table of hundreds of numbers is not easily understood. Heatmaps have become the standard method or presenting large data sets of gene expression across multiple samples. Furthermore, clustering algorithms have been developed in order to find and display any patterns that might remain hidden in the data [14]. A script written in the R programming language [15] generates two heatmaps of the miRNA expression data: one with the samples and miRNAs arranged in the order given present in the input, and another upon which unsupervised hierarchical clustering based on the Euclidean distances between samples and miRNAs has been performed. The normalized data used for the heatmap is also preserved as an output file, suitable for further statistical analysis. Together, these scripts allow patterns in the data to be visualized and hypotheses about differences between samples to be generated.

SMiRK also performs automated statistical analysis of the data. If two groups of samples are specified, then SMiRK will compare the normalized expression levels of every non-low-abundance miRNA in those two samples. For each miRNA, the Wilcoxon test [16] is used to calculate p-values and determine if a statistically significant difference in expression of that miRNA exists. Since the Wilcoxon test is non-parametric, it can robustly test groups of samples without relying on any assumptions about their distribution.

A final feature of SMiRK is its modularity and transparency. The output of each step is used as the input for each subsequent step, and the intermediate files generated are preserved for subsequent analyses. Although beginning users may only wish to use the final output of the entire SMiRK pipeline, more advanced users may desire more granular control over the processing and analysis of the data. After running SMiRK, users may inspect and modify the intermediate files and have the option of running modules of SMiRK individually. For example, a user may wish to arrange the samples in a configuration different than that produced by the unsupervised hierarchical clustering that SMiRK uses by default. That user could take the data from SMiRK after it had been normalized and low-abundance miRNAs removed, rearrange the samples and miRNAs with their desired method, and then run the heatmap generation module of SMiRK on the data. Although this method would be more involved than just running SMiRK, the pre-existing tools provided would make it much easier than manually implementing all of the steps. In this way, SMiRK allows investigators to choose how involved they are in data analysis. Beginning users can easily run SMiRK and make use of its output; but as they become more experienced, they can also examine the process and intervene if they wish.

## Results

**Figure 2:**
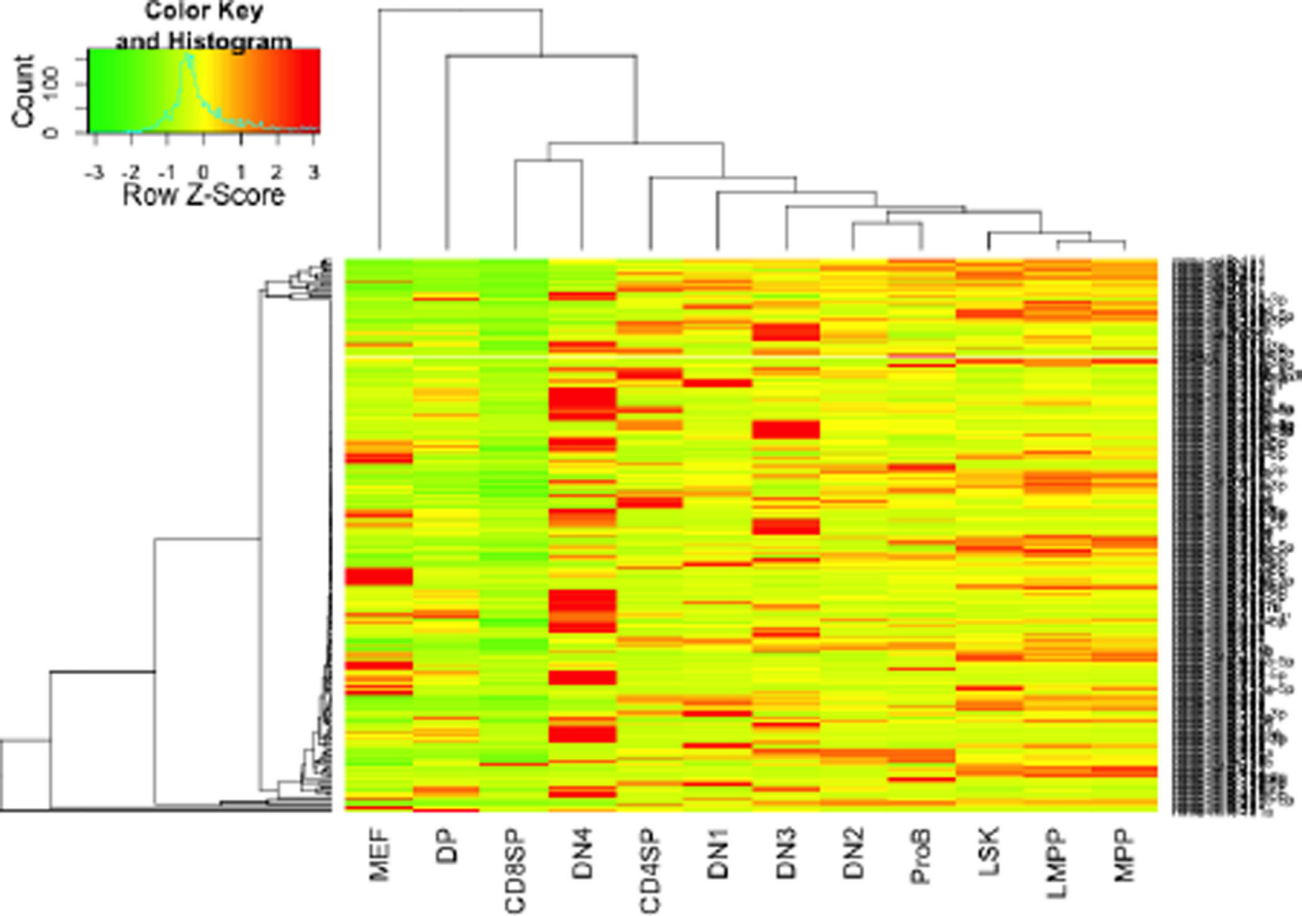
Heatmap generated by SMiRK.

As a proof of concept, SMiRK was run on a dataset of miRNA sequences from human T cells, pro-B cells and MEFs, previously reported by Kirigin et al. [17]. The data consisted of 23,830,788 miRNA reads divided among 12 samples, one from each cell type: ProB, LSK, MPP, LMPP, DN1, DN2, DN3, DN4, DP, CD4SP, CD8SP, and MEF The normalization and analysis took less than a minute to complete on a standard modern home computer. A heatmap (Figure 2) was produced. The heatmap shows that the pro-B cells cluster with the T cells, which is expected because they are related cell types. Furthermore, the MEFs were clustered away from the other cell types, which are also expected since they are unrelated cell types. The samples in the first half of T cell development (cLSK, MPP, LMPP, DN1, and DN2) and the second half of T cell development (DN3, DN4, DP, CD4SP, and CD8SP) were chosen as the two groups to compare. Of the 215 miRNAs with sufficient abundance to warrant further analysis, 39 had a nominal p-value of less than.05, highlighting them as candidates for genes involved in T cell maturation. These included mir-10a and mir-126-3p, which had been identified in the original study as being downregulated in later stages of T cell development; thus, SMiRK recapitulated previous findings from the dataset, demonstrating its reliability in detecting biologically relevant changes in miRNA expression. These final analysis outputs demonstrate SMiRK’s ability to detect patterns in data and display them in an easily understood format.

After processing the miRNA sequence data, SMiRK generates a heatmap of the data in order to produce an accessible and informative visualization. Unsupervised hierarchical clustering of both the samples and miRNAs is performed. In this heatmap, related cells types have been clustered together, with the one unrelated cell type, MEF, occupying a position on the dendrogram distal to all other samples.

## Discussion

In this paper, we describe SMiRK, a pipeline for rapid processing and analysis of data from high-throughput miRNA sequencing experiments. Features of SMiRK include adaptor trimming, alignment, normalization, data visualization, and statistical analysis. The main advantage of SMiRK is that it performs analysis of data without requiring the dedicated efforts of a bioinformatician or statistician. Furthermore, its highly automated nature means that little human intervention is needed to direct the data analysis, but the program can still prove useful to skilled users who wish to use it as a tool set. Web-based tools are generally low-throughput and their workflow cannot be easily modified or interfaced with other programs. Other locally installed tools, such as CAP-miRSeq [18], are designed for use on a cluster environment; these environments have high requirements in terms of both computing hardware and user knowledge. Our hope is that SMiRK will present a low barrier to entry for novice users, while also providing a path to greater knowledge and familiarity with the analysis being performed, as well as a set of tools useful for advanced users. Regardless of the investigator, using SMiRK greatly reduces the complexity and time investment involved in analysis of miRNA sequencing data. SMiRK provides a high-throughput analysis pipeline to match the high-throughput nature of sequencing technology.
